# EEG Findings in Infants With Neonatal Abstinence Syndrome Presenting With Clinical Seizures

**DOI:** 10.3389/fped.2019.00111

**Published:** 2019-03-29

**Authors:** Murali Reddy Palla, Gulam Khan, Zahra M. Haghighat, Henrietta Bada

**Affiliations:** Division of Neonatology, Department of Pediatrics, University of Kentucky, Lexington, KY, United States

**Keywords:** seizures, neonatal abstinence syndrome, electroencephalography, prenatal opiate exposure, sleep myoclonus

## Abstract

Neonatal abstinence syndrome (NAS) refers to a constellation of signs occurring in newborn infants who were exposed to opioids or opiates *in utero*. These manifestations include poor feeding, gastrointestinal disorders, abnormal sleep patterns, and neurological signs such as jitteriness, tremors, and seizures (1, 2). Myoclonus, jitteriness, and tremors often may be interpreted as seizures and therefore treated as epileptic seizures.

**Objective:** To determine whether seizure like activity observed in infants with NAS correlate with electroencephalogram (EEG) findings.

**Design/ Method:** We reviewed the standard EEG or video electroencephalogram (VEEG) of infants with NAS who were admitted because of seizure-like clinical activity. The exclusion criteria were major neurological anomalies, hypoxic ischemic encephalopathy, metabolic disorders, or with clinical diagnosis other than NAS.

**Results:** Forty neonates met study criteria; 28 had standard EEG recordings and 18 had VEEG. Mean gestational age was 38.5 weeks. The onset of seizure-like clinical activity was as early as day 1 and as late as day 16 of life. The clinical seizure-like activity described at the referring hospital were jerking, rhythmic movement of the extremities, or tremors. Only three (7.5%) neonates had epileptic seizures. There were increased sharp transients in frontal, central, temporal, and or occipital regions. VEEG showed disturbed non-rapid eye movement (REM) sleep with frequent arousal, jittery movements, or sleep myoclonus.

**Conclusion:** Clinical seizure-like activity correlates poorly with epileptic seizures in infants with NAS. In neonates with NAS, a VEEG would be useful to determine if the clinical seizure-like activity is of epileptic origin or not, prior to initiation of anti-seizure medications.

## Introduction

Neonatal abstinence syndrome (NAS) is a clinical diagnosis, and a consequence of the abrupt discontinuation of chronic fetal exposure to substances, specifically opiates or opioids used by the mother during pregnancy. The prolonged half-life of opioids in the infants and the ease with which opioids can cross the fetal blood-brain barrier of the fetus may increase the severity of withdrawal manifestations in infants with NAS (3). Furthermore, accumulation of the drug in the fetus may result in the increased production of various neurotransmitters through a cascade of enzymatic activities (4). Locus coeruleus is the most important center of activity in opioid withdrawal. This is the principal noradrenergic nucleus of the brain and is extremely sensitive to opioid status (5), i.e., withdrawal of the inhibitory effect of opiate on the adrenergic neurons results in the hyperactivity of these neurons (6). Most infant with NAS manifests with central nervous system irritability, autonomic over reactivity, and gastrointestinal tract dysfunction. Central nervous system manifestations reported with NAS are tremors, exaggerated Moro reflex, hypertonia, and myoclonic jerks (7). These can mimic seizures and therefore, an EEG may be required for confirmation prior to treatment.

Of neonates with NAS, 2–11% manifest with seizures (8). The underlying cause of seizures in NAS is not known, although the threshold for seizure activity may be decreased due to upregulation of sodium channels as a result of receptor instability (9). NAS may involve a short intense initial phase, consisting of tremors, seizures, irritability, feeding problems, vomiting, diarrhea, hyperthermia, and other systemic signs which lasts for upto 1–2 weeks. Later initial phase may be followed by a long chronic and relapsing course that includes hyperirritability, sleep disturbances, hyperphagia, and other neurologic and autonomic signs (lasting for a few weeks to a few months) (10). NAS symptoms depend on the type of drug exposure. In intrauterine opioids exposure, such as heroin is associated with earlier onset and shorter duration of withdrawal, compared to methadone or buprenorphine, which is associated with late onset and prolonged withdrawal. In the nonopioids class, methamphetamine exposure is associated with early withdrawal symptoms (11).

The jerking movement in NAS may be interpreted as seizures, thus an indication for anti-convulsant therapy. However, these jerking movements may represent benign neonatal sleep myoclonus (BNSM), which has been reported in neonates with NAS (1). Benign neonatal sleep myoclonus (BNSM) is a phenomenon typically observed in newborns during the first 4 weeks of life. This condition is not associated with abnormal EEG. BNSM does not need treatment and does not affect neurodevelopmental outcome (12–14).

Since clinical observation of myoclonus, jitteriness, or tremors can be misinterpreted as epileptic seizures, it prompted us to explore the association between myoclonus, jitteriness, tremors, and or seizure-like activity and EEG findings in babies with NAS.

## Methods

This retrospective study had approval by the Institutional Review Board (IRB) and met compliance with the Health Insurance Portability and Accountability Act. The IRB waived informed consent procedure for this study. Inclusion criteria included admission to the neonatal intensive care unit, history of intrauterine opioid exposure with or without other substances, seizure-like activity reported as a clinical manifestation, and the infant having had routine EEG or VEEG. Opioid exposure refers to a mother admitting use during pregnancy or had a positive urine test for opioids or the baby had positive urine or meconium drug test. Seizure-like manifestation was described as jerking or rhythmic movement of extremities, myoclonus during sleep, apnea, and/or tremors. The exclusion criteria were major neurological anomalies, hypoxic ischemic encephalopathy, metabolic disorders, or with clinical diagnosis other than NAS. Interval assessment or monitoring of these infants used the Finnegan Neonatal Abstinence Scoring System (2). Oxygen saturation monitored by pulse oximetry, blood glucose; sodium, potassium, and calcium were monitored. All infants were monitored with Finnegan scoring system. If 3 consecutive scores were ≥8 or 2 consecutive scores of ≥12, infants were treated with morphine escalated and weaned according to the unit protocol. If infants had electrographic seizures, they were treated as per our neonatal intensive care unit (NICU) guidelines.

The EEG and VEEG were reviewed by one of the investigators (GK), a pediatric epileptologist. Routine EEG was done for 1 h and VEEG was done for 48 h. The Xltek EEG/PSG System (Natus Neuro Inc., Pleasanton, CA) was used for both EEG and VEEG; it has components needed for EEG and VEEG. The EEG and VEEG waveforms were assessed for the background activity, sharp transients, and electrographic seizures. Background activity is considered normal if there is no focal slowing and no prolonged “trace alternant” (in term neonates 6–8 s is normal). Sharp transients are normally present in newborn infants but disappear by 50 weeks of post conceptional age. Sharp transients that are repetitive in same area with slow background and increased frequency are considered abnormal (15). Neonatal electrographic seizure refers to a sudden, repetitive, evolving, and stereotyped event of abnormal electrographic pattern with amplitude of at least 2 μV and a minimum duration of 10 s (16). Descriptive statistics were used and no EEG scoring system was used.

## Results

Forty neonates with NAS were admitted to our level IV neonatal intensive care unit (NICU) for seizure-like manifestation during January 2010- November 2015. The patient characteristics are shown in Table 1. There were 23 males and 17 females. The mean (SD) gestational age was 37.5 (1.9) weeks and mean (SD) birth weight was 2,843 (620) grams. All infants were exposed to tobacco during pregnancy and of whom 29 infants were exposed to opioids only. The remaining 11 infants were exposed to more than one drug (marijuana, benzodiazepines, methamphetamines, and/or gabapentin).The onset of seizure-like manifestation was as early as day 1 and as late as day 16 of life, mean (SD) of 4.1 (4) days. Twenty-seven neonates were treated for seizure-like clinical sign at the referring hospital before EEG was performed; 23 were treated with phenobarbital, two with morphine, and one with levetiracetam. In these patients, seizure like clinical activity were described as jerking or rhythmic movement of extremities, myoclonus during sleep, apnea, and/or tremors. Based on review of the EEG or VEEG, only three neonates had epileptic seizure. Findings on EEG are shown in Table 2. Most of the neonates (72.5%) had normal background activity and 27.5% had mildly slow activity. Three neonates (7.5%) with epileptic seizures also had mildly slow background activity. There were sharp transients in frontal, central, temporal, and or occipital regions. Additionally, in the 18 (45%) neonates who had continuous video monitoring, detailed review showed disturbed non-REM sleep with frequent arousal pattern, jittery movements, and sleep myoclonus. BNSM was seen in 13/18 (72%) neonates and 5/18 (28%) had increased arousal pattern. Oxygen saturation monitored by pulse oximetry, blood glucose, sodium, potassium, and calcium were within normal limits (Table 3).

**Table 1:** Patient characteristics.

Total number of subjects	40
Males	23 (57.5%)
Females	17 (42.5%)
Mean gestational age (SD)	37.5 weeks (1.9)
Mean birth weight (SD)	2,843 g (620)
Intrauterine exposure to single drug	29 (72.5%)
Intrauterine exposure to poly drugs	11 (27.5%)
Day of life onset of reported seizure, mean (SD)	4.1 (4)
Day of life onset of reported seizure, median (range)	2.5 (1–16)
Number of Pts treated for clinical seizures with anti-seizure medications before admission/EEG	23 (57.5%)
Not treated for clinical seizures	17 (42.5%)

**Table 2:** Electroencephalographic findings in infants with neonatal abstinence syndrome observed to manifest with clinical seizures.

**Routine EEG and VEEG (*n* = 40)**	***n* (%)**
**BACKGROUND ACTIVITY**	
Normal	29 (72.5)
Mildly slow	11 (27.5)
**SHARP TRANSIENTS**	
Fronto- centro-temporal (FCT)	21 (52.5)
Fronto centro temporal occipital (FCTO)	8 (20)
Centro temporal (CT)	7 (17.5)
Centro temporal occipital (CTO)	2 (5)
No transient spikes	2 (5)
**SEIZURES**	
No seizures	37 (92.5)
Seizures present	3 (7.5)
**VEEG (*****n*** **=** **18)**	
Benign neonatal sleep myoclonus (BNSM)	13 (72)
Increased arousal pattern	5 (28)

**Table 3:** Oxygen saturations and biochemical parameters on admission.

**Parameters**	**Mean and standard deviation (SD)**
Oxygen saturations, %	96.65 (1.57)
Blood glucose, mg/dL	81 (16.69)
Sodium, mmol/L	139 (3.38)
Potassium, mmol/L	4.7 (0.72)
Calcium, mmol/L	9.4 (0.94)

## Discussion

We report EEG findings in neonates with NAS, who were admitted for clinical seizure like activity. The neurological examination was normal, except for the irritability, tremors, and increased tone, which are not unusual findings in babies with NAS.

Herzlinger and co-investigators reported an incidence of 5.9% of documented seizures in neonates with NAS (8). In our study, of infants with NAS who presented with seizure-like activity, majority (92.5%) had no electroencephalographic seizures; only three or 7.5% had seizures consistent with the EEG pattern. Our findings suggest that seizures if confirmed by EEG studies occur at a much lower incidence than in previous reports based on clinical findings. Our findings on EEG were consistent with disturbed non-REM sleep with frequent arousal, Jittery movements, or sleep myoclonus (1, 17). Benign neonatal sleep myoclonus or (BNSM) is a distinctive disorder characterized by rhythmic myoclonic jerks, which occur when the child is asleep and stop when the child is awakened. BNSM may appear on the first day of life and persist for several weeks to months. BNSM is often mistaken for epilepsy but the EEGs are always normal. The pathophysiological mechanism of BNSM is not fully elucidated (18). It may a result of a benign discharge in the reticular activating system, where the initiation and synchronization of normal sleep is controlled (19). Another possibility is that BNSM is related to transient immaturity or imbalance of the serotoninergic system (20). Because of the spontaneous resolution with age, BNSM it is likely related to brain maturation. Previously reported clinical seizures in NAS could at least in part have been myoclonic jerks (12). An earlier study showed around 67% of infants with NAS and had EEG procedure had BNSM (1). In our study of 18 neonates with VEEG, BNSM was seen in a comparable incidence (13/18 or 72%). Five or 28% had increased arousal pattern. In children and adults, the most specific interictal electrographic signs of the seizure activity are spikes and sharp waves. The neonatal situation is more complex, since some sharp transients are commonly seen in their EEG (21). Further, in healthy neonates, sharp EEG transients usually appear against a normal background and which do not signify the presence of encephalopathy nor support the presence of seizures. The clinical significance of sharp wave transients on neonatal electroencephalographic (EEG) recordings remains controversial (22). Rowe et al. stressed that EEG background abnormalities are a more accurate independent predictor of neurodevelopmental outcome rather than sharp wave transients. In our study there were increased sharp transients in frontal, central, temporal, and occipital regions, but with normal background activity (23). EEG background activity was normal in 72.5% of our patients; none of them had electrical seizures. Eleven or 27.5% had mildly slow pattern background; the three with electrical seizures were among those with mildly slow background activity.

Few studies examined sleep in NAS. Opiate-exposed newborns have shown a significant increase in active sleep and a reciprocal decrease in quiet sleep than controls (23–26). O'Brien and Jeffery reported on a reduction of quiet sleep in infants with NAS compared to their control group (8). Also, adult and preclinical studies demonstrated that sleep is altered in opiate withdrawal (27–30). The mechanisms behind sleep disturbances during NAS remain unclear, but could be due to changes to the central nervous system (CNS) during opiate dependency *in utero*, and due to hyperactivity of the CNS during withdrawal (23, 25). In our study, the neonates who had VEEG, 28% did have increased arousal pattern.

## Limitations

Our study merely focused on infants with NAS admitted for seizure-like clinical manifestation. VEEG was not performed in all study subjects. The small number of infants in our study precluded analysis of any correlation between the EEG findings and the type of opioids or other drugs to which the infants were exposed. The impact of medication used to treat NAS (or seizures) on the EEG was not analyzed.

## Conclusion

Clinical manifestation of seizure-like activity correlates poorly with epileptic seizures in infants with NAS. In infants with a history of intrauterine drug exposure mainly opioids presenting with seizure like activity with no other medical conditions, obtaining a video EEG would be ideal to determine if the seizure-like manifestation is of epileptic origin, before starting anti-seizure medication. If available, a VEEG is a preferred modality to be able to correlate abnormal movements with electroencephalographic findings.

## Ethics Statement

Study was approved by University of Kentucky Institutional Review Board.

## Author Contributions

MP reviewed the charts, EEG along with pediatric neurologists. ZH did the chart review and EEG review. GK did review of EEG's. HB guided through the project.

### Conflict of Interest Statement

The authors declare that the research was conducted in the absence of any commercial or financial relationships that could be construed as a potential conflict of interest.
